# Prostatic Stromal Tumor of Uncertain Malignant Potential Which Was Difficult to Diagnose

**DOI:** 10.1155/2015/879584

**Published:** 2015-12-29

**Authors:** Satoko Matsuyama, Takahiro Nohara, Shohei Kawaguchi, Chikashi Seto, Yuko Nakanishi, Akio Uchiyama, Shin Ishizawa

**Affiliations:** ^1^Department of Urology, Toyama Prefectural Central Hospital, 2-2-78 Nishinagae, Toyama, Toyama 930-8550, Japan; ^2^Department of Pathology, Toyama Prefectural Central Hospital, 2-2-78 Nishinagae, Toyama, Toyama 930-8550, Japan

## Abstract

Here, we report a case of stromal tumor of uncertain malignant potential (STUMP) that was difficult to diagnose. A 53-year-old male was found to have a hard nodule on digital rectal examination; magnetic resonance imaging revealed a large nodule on the left side of the prostate, indicating prostate cancer. However, pathological diagnosis of the biopsy specimen was benign prostatic hyperplasia. Although a papillary tumor in the prostatic urethra was also seen on urethrocystoscopy, the tumor specimen obtained from transurethral resection was not malignant. The tumor in the prostatic urethra recurred only 3 months after transurethral resection, and pathological findings revealed benign hyperplasia not only in the stromal tissue but also in the epithelium; therefore, the prostate tumor was suspected to be STUMP. It took many prostate pathologists a long time to reach the final diagnosis of STUMP. STUMP is a rare benign tumor, difficult to diagnose, and sometimes transforms into stromal sarcoma. Thus, we should consider radical resection in such cases.

## 1. Introduction

Stromal tumors of uncertain malignant potential (STUMP) are rare tumors characterized by an atypical, unique stromal proliferation of the prostate. The pathological diagnosis of STUMP is sometimes confused with the stromal proliferations present in BPH, and the distinction may be difficult [1]. Here, we describe a case of STUMP that was difficult to diagnose.

## 2. Case Presentation

A 53-year-old male was suspected of having prostate cancer because of elevated prostate-specific antigen (PSA) levels at screening. He underwent transrectal biopsy of the prostate at a nearby clinic, which revealed no evidence of malignancy.

A year later, he came again to the clinic with the symptom of macroscopic hematuria. The PSA level was 14.7 ng/mL (normal, <4.0 ng/mL). Digital rectal examination revealed a hard nodule in the left lobe of the prostate, and cystoscopy revealed a papillary tumor in the prostatic urethra. Transrectal biopsy of the prostate and transurethral resection (TUR) of the tumor in the prostatic urethra were performed. The pathological diagnosis was “ductal adenocarcinoma of the prostate” from the specimen obtained from TUR.

The left obturator lymph node was slightly enlarged on computed tomography, and the clinical stage was determined to be T3bN1M0. Androgen deprivation therapy (ADT) by bicalutamide was started. Thereafter, he was referred to our institution for radiotherapy.

**Figure 1:**
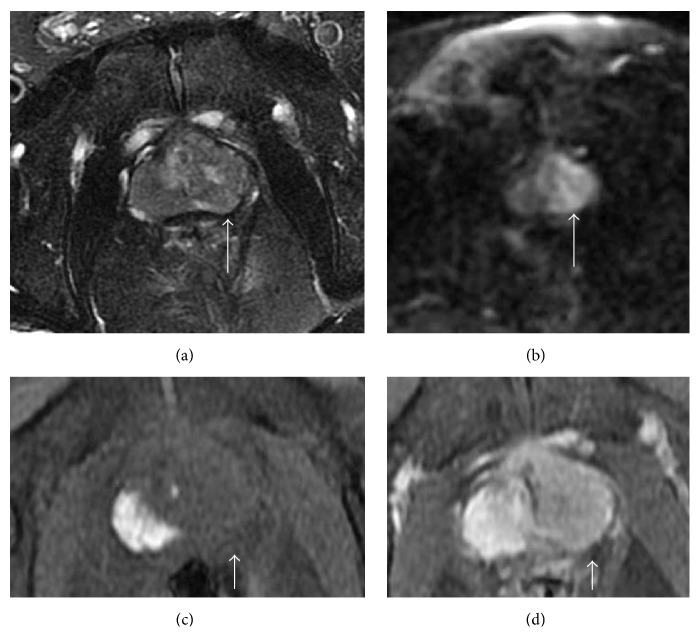
On MRI, T1 weighted image showed diffusely spread tumor in the left side of the prostate (white arrow). This lesion was heterogeneous on T2 weighted images (a), high intensity on diffusion weighted images (b), and well enhanced by contrast agent ((c) T1 weighted image, (d) contrast-enhanced T1 weighted image).

On magnetic resonance imaging, there was a large spread nodule on the left side of the prostate without any signs of seminal vesicle invasion (Figure 1). These clinical findings confirmed the diagnosis of prostate cancer. However, our pathological diagnosis of the biopsy specimen brought from the clinic was different. We diagnosed benign prostatic hyperplasia (BPH) with no evidence of malignancy because there were no findings suggesting prostate cancer such as nuclear enlargement and dyskaryosis in the epithelial cells. A repeat transrectal prostate biopsy also indicated BPH. The discrepancy between clinical and pathological findings was confusing. However, based on the results of the pathological examination, we withdrew the diagnosis of ductal adenocarcinoma (T3bN1M0). We stopped bicalutamide and canceled the plan of radiotherapy.

**Figure 2:**
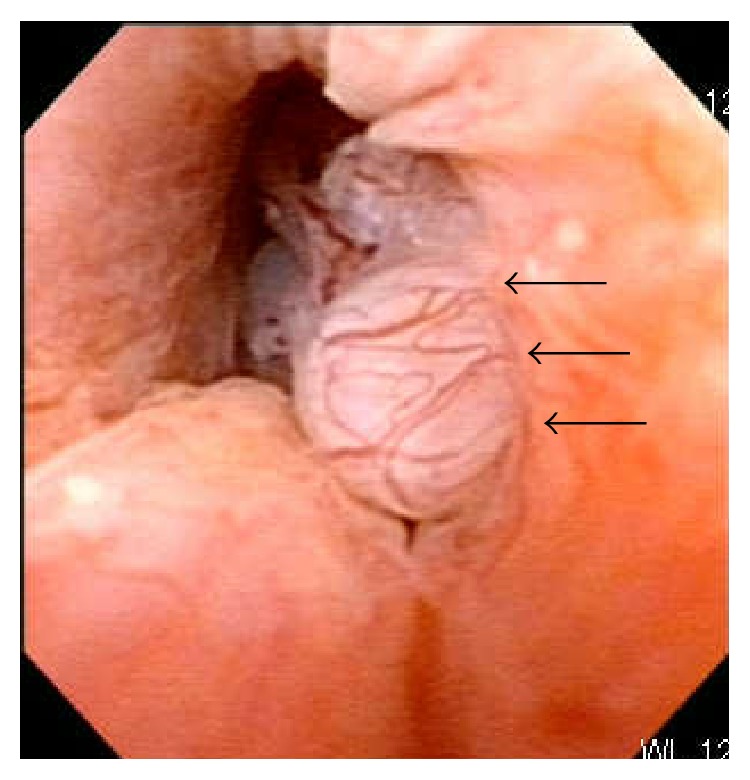
Cystourethroscopy before TUR. It revealed that a papillary tumor was in the left side of the prostatic urethra with mild inflammatory change on the mucosa (black arrows). This finding was almost the same as the findings of the figure which was pictured at the time of cystourethroscopy in the clinic he had visited at first.

**Figure 3:**
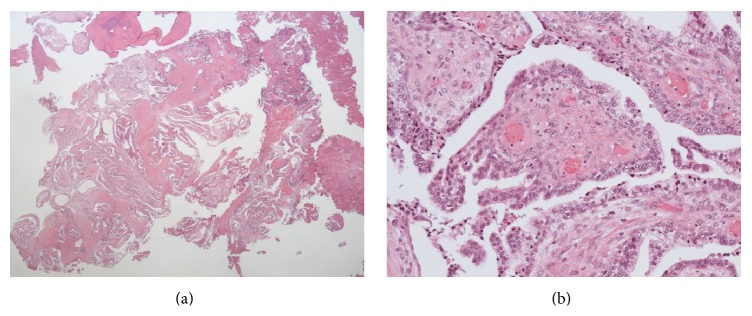
Pathological findings of TUR ((a) ×40, (b) ×400). There was benign hyperplasia not only in the stromal tissue but also in the epithelium, without nuclear enlargements. It was just like a phyllodes tumor in the mammary gland.

During a follow-up visit 6 months later, the patient complained again of macroscopic hematuria. Cystoscopy revealed a papillary tumor in the prostatic urethra with mild inflammatory changes in the mucosa, which was similar to the information obtained from the nearby clinic (Figure 2). We performed TUR again and resected most of the tumor. Pathological examination revealed benign hyperplasia not only in the stromal tissue but also in the epithelium (Figure 3). These findings were very similar to a phyllodes tumor of the breast. Because it seemed to be a very rare tumor, we decided to consult experts on prostate pathology in Japan. Several prostate pathologists discussed the diagnosis for a long time, but they could not confirm the pathological diagnosis; they could only suspect STUMP.

**Figure 4:**
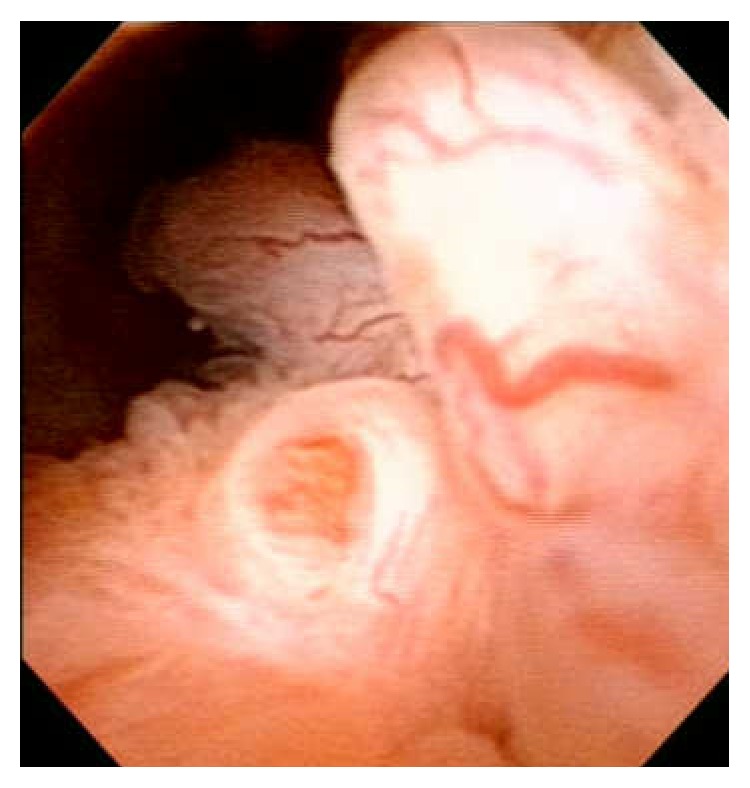
Urethrocystoscopy 3 months after TUR. It revealed that a papillary tumor was there again in the left side, with the prostatic urethra opened. This finding was almost the same as the tumor before TUR.

**Figure 5:**
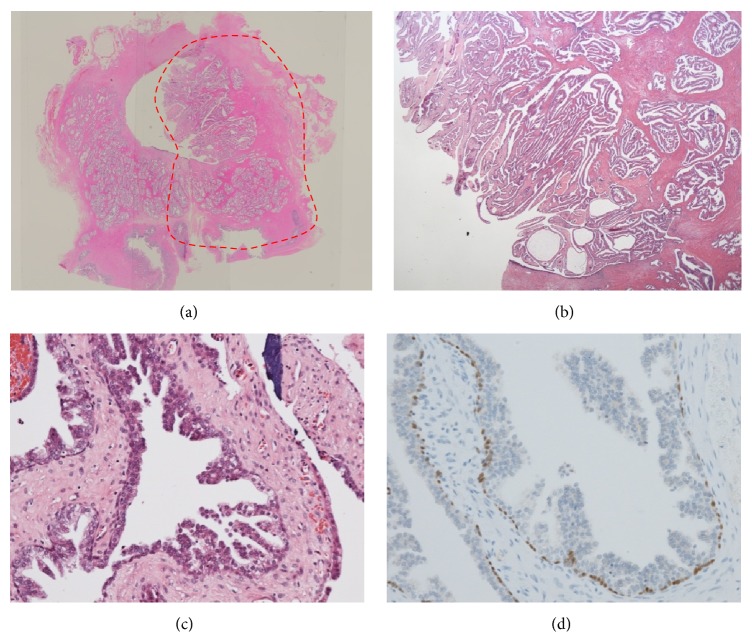
Pathological findings of the specimen obtained from radical prostatectomy ((a) ×4, (b) ×40, and (c) ×400). The pathological finding was that epithelial cell proliferations without nuclear enlargement were frequently seen in the tumor of the prostate. It was just like benign epithelial hyperplasia and there was no evidence suggesting malignant tumor. Immunohistochemical pathology revealed that basal membranes were preserved by p63 staining and few cells were stained by p504S (d), suggesting that the tumor was not malignant.

Follow-up cystoscopy at only 3 months after TUR revealed recurrence of the large papillary tumor at the same site (Figure 4). We believed that repeat TUR would only result in early recurrence and increase the risk of conversion to stromal sarcoma (SS) if the tumor was indeed STUMP; therefore, we performed radical prostatectomy and pelvic lymph node dissection. The operative time was 300 minutes, and the total intraoperative bleeding was 1400 mL. The pathological findings revealed epithelial cell proliferations without nuclear enlargement, resembling benign epithelial hyperplasia; there was no evidence suggesting malignancy (Figure 5). It took a long time to establish the final diagnosis because it was difficult to diagnose; we had to consult specialists on prostate pathology in Japan and the United States. They concluded that the final diagnosis was prostatic STUMP with a feature of epithelial proliferation. There were no SS and lymph node metastasis. Complications did not occur, and there has been no evidence of metastasis or recurrence for 4 years after radical prostatectomy.

## 3. Discussion

STUMP is a rare tumor characterized by an atypical, unique stromal proliferation of the prostate. Previously, STUMP had been reported in a variety of ways including atypical stromal (smooth muscle) hyperplasia, phyllodes type of atypical stromal hyperplasia, and cystic epithelial-stromal tumors. In 1998, these tumors were called STUMP, when Gaudin et al. [2] reported a series of 22 cases with specific histological and immunohistochemical features that were distinct from prostatic SS. The phrase “uncertain malignant potential” was employed to describe the diversity of biological behavior.

STUMP shows various features of progression. A retrospective study suggested that the tumor recurred in 46% of cases in patients who did not undergo complete resection [2]. In some cases, the tumor recurs several times and requires multiple procedures over time [3]. Furthermore, STUMP sometimes transforms to SS and metastasizes most commonly to the lung and lymph nodes. Two of the three patients in these reports died of SS [4–6].

The pathological diagnosis of STUMP is sometimes very difficult, even by pathologists specializing in prostate pathology. STUMP can be histologically misdiagnosed as BPH. It is more difficult to distinguish BPH from STUMP when only small specimen is obtained such as prostate biopsy. However, it is important to recognize that STUMP often recurs and has the potential to change to SS. STUMP can occur at a younger age than BPH, predominantly involve the peripheral zone, and often adhere to the rectum [3]. Therefore, close follow-up may be required for patients with BPH who are young and have recurrence early after surgery, even though the pathological diagnosis is BPH. Furthermore, complete extraction should be considered in patients with STUMP.

In the present case, it took a long time to establish the final diagnosis because there was proliferation not only in the stromal tissue but also in the epithelium. At first, the pathologist of the other institution (not specialized in prostate pathology) diagnosed ductal adenocarcinoma. We thought that he made a mistake because remarkable epithelial proliferation was seen in the specimen. We corrected the diagnosis to BPH because there were no findings suggesting prostate cancer such as nuclear enlargement and dyskaryosis in the epithelial cells. However, we had to consider that the tumor might have some malignant feature because of the clinical findings such as rapid growing and MRI findings. We suspected STUMP at this time. However, benign epithelial proliferation made it very difficult for us to diagnose STUMP because STUMP is normally recognized as having proliferation of stromal tissue. We had to consult specialists on prostate pathology in Japan and the United States, and we conclude that the diagnosis was “STUMP with a feature of epithelial proliferation” according to their opinion.

Before radical prostatectomy, the diagnosis of STUMP was only suspected. However, radical prostatectomy seemed to be a better option than repeat TUR because the tumor in the prostate (suspicious of STUMP) had a tendency of rapid growth and was not amenable to complete resection by TUR, and such a procedure could transform the tumor from STUMP to SS.

In summary, we report a case of prostatic STUMP that was difficult to diagnose. STUMP is rare and can be histologically misdiagnosed as BPH. It has the potential to transform into SS. Therefore, STUMP should be managed aggressively with radical resection.
